# Spatial Spillover Effect of Rural Labor Transfer on the Eco-Efficiency of Cultivated Land Use: Evidence from China

**DOI:** 10.3390/ijerph19159660

**Published:** 2022-08-05

**Authors:** Xiuqing Zou, Meihui Xie, Zhiyuan Li, Kaifeng Duan

**Affiliations:** 1 College of Economics and Management, Shanghai University of Electric Power, Shanghai 201306, China; 2 School of Economics, Jiangxi University of Finance and Economics, Nanchang 330013, China; 3 Institute of Ecological Civilization, Jiangxi University of Finance and Economics, Nanchang 330013, China; 4 School of Economics and Management, Fuzhou University, Fuzhou 350108, China

**Keywords:** rural labor transfer, eco-efficiency of cultivated land use (ECLU), undesirable super-efficiency EBM model, spatial Durbin model (SDM), spatial spillover effect

## Abstract

In this study, the influence of rural labor transfer and its spatial spillover effect on the eco-efficiency of cultivated land use (ECLU) in different regions were investigated using the undesirable super-efficiency epsilon based measure (EBM) and spatial Durbin models and data of 31 Chinese provinces for the period 1990–2018. The results show that: (1) China’s rural labor transfer rate increased; (2) in the east region, the ECLU has exceeded the national average level since 2001. In the west and northeast regions, the ECLU was higher, whereas it remained below the national average level in Central China; (3) in the whole country, west, and northeast regions, the effect of rural labor transfer on the ECLU was first negative and then positive, whereas it was insignificant in East and Central China. In Central, West, and Northeast China, the effect of the labor transfer on the ECLU had significant spatial spillover effects; (4) a significant U-shaped trend was observed between the local labor transfer and ECLU in the whole country, west, and northeast regions. A positive linear correlation was determined for Central China; labor transfer in other regions had significant indirect effects on the ECLU in Central and Northeastern China. In conclusion, China’s rural labor transfer had a significant spatial spillover effect on the ECLU, and differences were observed between East, Central, West, and Northeast China.

## 1. Introduction

Rural labor transfer is an important factor affecting the economic development and alleviation of rural poverty. The mode and process of labor transfer is different due to different economic situations, cultural and historical backgrounds, and stages of development. Britain, the United States, Canada, Japan, and other developed countries at the end of the 20th century basically completed the rural labor transfer, but most developing countries are still in the continuous stage of rural labor transfer. As the largest developing country, the number of rural labor transfer increased from 87.94 million to 139.09 million, and the proportion of agricultural rural labor decreased from 60.1% to 26.1% from 1990 to 2018 in China, but it is still far from the ideal proportion of 10% (Source: National Bureau of Statistics of China). Rural labor is one of the key elements of agricultural production; its transfer inevitably changes the allocation of labor, land, capital, and other elements in the agricultural production system [1,2,3,4,5]. Rural labor transfer affects the technical, scale, and resource allocation efficiencies of cultivated land use, and, subsequently, the eco-efficiency of cultivated land use (ECLU) [6]. Due to factors such as the cross-regional flow of labor, capital, technology and other elements, such as the complete non-exclusivity of the ecological benefits of cultivated land use [7] and the adjacent spatial location of cultivated land, the behaviors of cultivated land use entities indirectly affect the welfare of other entities [8]. Based on the concept of the “spillover effect or externality” in economics [9], New Economic Geography refers to the effect of the behavior of a regional economic entity on other regional entities as spatial spillover [10,11]. The negative externalities of cultivated land use, positive externalities of knowledge and technology spillovers, and positive effects of scale operation due to the “transfer” of nonpoint source (NPS) pollution based on the cultivated land use of local farmers [12] have non-negligible effects on the cultivated land use efficiency in neighboring areas. Although China uses less than 20 percent of the world’s arable land to feed more than 20 percent of the world’s population, it also pays a huge cost of resources and the ecological environment. Hence, exploring the influence of rural labor transfer and its spatial spillover effect on the ECLU is of great significance for increasing the ECLU in China and other developing countries, guiding cultivated land use behaviors in different regions, and ensuring global food security and social stability.

In most previous studies, data envelopment analysis (DEA) [13] and stochastic frontier approach (SFA) [14] models were used to calculate the technical efficiency of cultivated land use and the slack-based measure (SBM) [15] and super-efficiency SBM [16] models were utilized to calculate the eco-efficiency. Based on the input–output perspective, selected indicators experienced a gradual-in-depth research process from “multiple inputs and single output” to “multiple inputs and multiple outputs,” then to “multiple inputs and multiple outputs” with consideration of environmental factors. In terms of factors affecting the efficiency, several scholars have studied the effect of labor transfer on the efficiency of agricultural production in China and Norway [17,18] and the spatial spillover effect of labor transfer on the county-wide efficiency of agricultural production in China [19]. Some researchers studied the overall spatial spillover effect of the labor transfer on China’s agricultural eco-efficiency [20]. However, there is a lack of studies at the regional level.

Based on the results of several studies in which the ecological environment was not considered, agricultural labor is negatively correlated with the cultivated land use efficiency [21]. The labor force per unit area of land significantly promotes the increase in the cultivated land use efficiency in Northeast China, but the situation is opposite in the central and western regions [22]. The contribution of labor transfer in the Beijing–Tianjin–Hebei region to the increase in the cultivated land use efficiency differs among development zones to be optimized, key development zones, main production areas of agricultural products, and ecological protection zones [23]. When environmental pollution and carbon emissions were considered, Shanghai, Jiangsu, and Zhejiang in the east benefit from the increase in the technical efficiency (TE). In these locations, the ECLU increased and the endowment of cultivated land resources, level of economic development, and government expenditure on agriculture were important factors influencing the spatial and temporal differentiation [24]. The ECLU of the main grain-producing areas, such as Hunan and Jiangxi, in the middle and lower reaches of the Yangtze River, slightly decreases annually and exhibits a positive spatial spillover effect of “high–high and low–low” aggregation [25]. Based on the use of the SFA model and the spatial externality, the labor transfer in China’s counties had a significant positive spatial spillover effect on the agricultural production efficiency from 2002–2010 [19]. In contrast, based on the use of the SBM model, China’s interprovincial agricultural eco-efficiency steadily increased, with fluctuations, and the labor transfer had a significant spatial spillover effect on the agricultural eco-efficiency; however, regional differences were not analyzed [20]. In addition, the cultivated land use efficiency was lower when environmental factors were considered [26]. When undesirable outputs were considered, for the EBM model included the substitution effect of input factors [27], the efficiency value of the SBM model was smaller than that of the EBM model. the results of the EBM model were in line with the actual situation [28].

Based on a literature review, few scholars used the super-efficiency EBM model to calculate the ECLU. However, the super-efficiency EBM model yields more accurate efficiency values. Research on the effect of China’s rural labor transfer on the ECLU is mostly limited to the short period of 2000–2016 and less attention has been paid to their correlation since 1990. In addition, the spatial externality of the ECLU itself and the influence of the spatial externality of China’s rural labor transfer on the ECLU were ignored in most studies. Labor is the main factor influencing the production and the labor transfer across regions and industries has significant spillover effects [29]. Rural labor transfer promotes the allocation of resources in local and adjacent regions through income, demonstration, and knowledge spillover effects, thereby indirectly affecting the ECLU of adjacent regions. If the spatial correlation and heterogeneity are ignored, it is difficult to effectively control the NPS pollution due to chemical fertilizers and achieve interregional ecological compensation based on the “who pollution who control” principle. Therefore, to implement the “two-type agricultural model” (resource-saving and environment-friendly agriculture) and sustainable development concept, we calculated the ECLU of 31 Chinese provinces (municipalities and autonomous regions) from 1990 to 2018 using the undesirable super-efficiency EBM model. We also explored the spatial spillover effects and regional differences of the impact of labor transfer on the ECLU using the spatial Durbin model (SDM) to provide a reference for the establishment of regional agricultural cooperation, determine the mechanism of cultivated land use, and promote the overall improvement of the regional ECLU.

The main contributions of this paper are as follows. Firstly, the influence mechanism of rural labor transfer on the ECLU is theoretically analyzed, which enriches the existing research results. Secondly, the undesirable super-efficiency EBM model is used to measure the ECLU, and then the correlation and heterogeneity of the ECLU in geographical space is explored, which is conducive to effectively controlling the source of non-point source pollution and carbon emission. Thirdly, the SDM is used to explore the spatial spillover effect and regional differences of rural labor transfer on the ECLU, which is conductive to clarifying the development law between different regions and providing reference for the establishment of the linkage mechanism between regional agricultural cooperation and cultivated land utilization.

## 2. Theoretical Analysis and Research Hypothesis

The input of labor force, capital, land, and other factors is the key factor affecting the benefit of cultivated land output. The transfer of rural labor force is a rational choice for farmers to avoid the “involution” of agricultural production and seek income maximization [30]. When the land elements remain unchanged, and with the continuous large transfer of the labor force, farmers need to reasonably increase the input of capital elements to keep the output of cultivated land unchanged. In the 1990s, due to the fragmentation of farmland and the low penetration rate of agricultural machinery technology, the replacement elasticity of chemical fertilizer to labor force is about three times that of machinery; increasing the input of capital factors such as chemical fertilizers and pesticides is the main way to ensure the output of cultivated land. However, the accumulated non-point source pollution caused by chemical fertilizers and pesticides and the excessive carbon emissions caused by inefficient agricultural production behaviors are not conducive to the improvement of the ecological efficiency of cultivated land utilization. With the marginal benefit of chemical fertilizers, pesticides and other element decreases, the popularization of agricultural machinery technology, and the rectification of basic farmland and water conservancy facilities, the use of modern technology for large-scale operation cannot only help rationally fertilize and scientifically produce, but also effectively help to reduce non-point source pollution and carbon emissions, so as to improve the ECLU by improving the scale efficiency and technical efficiency of cultivated land utilization [31].

Moreover, based on the concept of “spillover effect or external effect” in economics [9], the new economic geography refers to the influence of the behavior of a regional economic subject on other regional subjects as spatial spillover [10,11]. Labor force is the main body of various economic activities, and the cross-regional and cross-industry transfer of labor force has a significant spillover dividend phenomenon. Rural labor force affects the willingness of farmers in other areas to transfer their cultivated land through informal channels such as social networks, thus affecting the scale of cultivated land operation and the adoption of agricultural machinery technology in other areas [32]. At the same time, in the process of cultivated land utilization and due to the existence of the income effect, demonstration effect and knowledge spillover effect, farmers’ resource allocation of local manpower, chemical fertilizers, pesticides, agricultural machinery, land and other factors will affect the resource factor allocation of farmers in other areas, thus changing the ECLU of other regions.

It is worth noting that the influence of labor transfer on the ECLU and its spatial spillover effect vary from region to region [33]. The per capita cultivated land in the eastern region is 0.65 mu (1 mu = 0.0667 hectares; source: 2019 China Environmental Statistics Yearbook), with a developed economy and technology. Non-agricultural employment income, employment channels and local employment convenience of the eastern region are better than in other regions. Farmers in the eastern region have a stronger willingness than other regions to transfer farmland, which is more conducive to mechanized large-scale production. Therefore, the impact of labor transfer on the ECLU in the eastern region may be smaller. Most of the labor force is transferred locally, and its efficient agricultural machinery production mode is difficult to promote in other regions in the short term, resulting in the smaller spatial spillover effect of the labor force transfer in the eastern region. The per capita cultivated land in the central region is nearly twice that of the eastern region. In the central region, due to the limited investment in agriculture, the farmland infrastructure in this region is not perfect, the progress of farmland leveling is slow, and the feasibility of using efficient agricultural machinery technology for production is small, which affects the development of large-scale agriculture. At the same time, the backward agricultural production mode and dense water network structure in the central region will lead to the transfer of chemical non-point source pollution, which will inevitably affect the level of farmland utilization in other surrounding areas. The per capita cultivated land in the west and northeast regions is 2.19 mu and 3.94 mu, respectively. Scale operation situation and the convenience of agricultural machinery operation are generally good in these regions. Due to the wide and sparse land, the reduction of agricultural labor force has an obvious impact on the scale utilization of cultivated land. Especially in the northeast region, the fertile soil is concentrated and contiguous; this feature cannot only save manpower, but also give full play to the advantages of agricultural machinery and equipment, and thus may have a crowding out effect on the surrounding areas, reducing farmers’ enthusiasm for low-carbon production, and may inhibit the improvement of the ECLU.

Based on the above theoretical analysis, the following research hypothesis is proposed:

**H1.**  *At the national level, the impact of labor transfer on the ECLU may be a gradual transformation process from negative to positive, and there may be a significant spatial spillover effect.*

**H2.**  *The influence of labor transfer on the ECLU in different regions may be different, and the spatial spillover effect varies from region to region.*

## 3. Materials and Methods

### 3.1. Research Methods

#### 3.1.1. Super-Efficiency EBM Model

At present, many scholars have used the SBM model to calculate the eco-efficiency. However, the projected point of the SBM is the farthest frontier point from the decision making unit (DMU), contrary to the closest point actually expected [34]. To make up for this shortcoming, Tone and Tsutsui [35] proposed a hybrid distance function EBM model:

(1) $$\begin{array}{c} min\frac{\theta -\varepsilon^{-}\left(1/\sum_{i=1}^{m}w_{i}^{-}\right)\sum_{i=1}^{m}\frac{w_{i}^{-}s_{i}^{-}}{x_{k}}}{\phi +\varepsilon^{+}\left(1/\sum_{r=1}^{q}w_{r}^{+}\right)\sum_{r=1}^{q}\frac{w_{r}^{+}s_{r}^{+}}{y_{k}}}\\ s.t.X\lambda -\theta x_{k}+s^{-}=0\\ Y\lambda -\phi y_{k}-s^{+}=0\\ \lambda \geq 0,s^{-}\geq 0,\theta \leq 1,\phi \geq 1 \end{array}$$

where λ is the linear coefficient of the DMU; *m* and *q* are the number of input and output indicators, respectively; $s^{-}$ and $s^{+}$ are input and output slack variables, respectively; θ is the radial efficiency value; w is the relative weight of each input indicator, satisfying ∑w = 1; and ε is a key parameter with a value range of [0, 1]. When ε = 0, the EBM model can be simplified to a radial DEA model; when θ = ε = 1, the EBM model will be changed to an SBM model.

Because the efficiency value of the EBM model is in the [0, 1] range, when the efficiency value is greater than 1, it is not able to observe. Andersen et al. [36] proposed a super-efficiency model to solve this problem:

$$min[\theta -\varepsilon \left(\overset{m}{\underset{i=1}{\sum }}s_{i}^{-}+\overset{s}{\underset{r=1}{\sum }}s_{r}^{+}\right)]$$

(2) $$s.t.\left\{\begin{array}{l} \overset{n}{\underset{j=1,j\neq k}{\sum }}\lambda_{j}x_{ij}+s_{i}^{-}=\theta x_{ik}\;(i=1,2,\cdot \cdot \cdot ,m)\\ \overset{n}{\underset{j=1,j\neq k}{\sum }}\lambda_{j}y_{rj}-s_{r}^{+}=y_{rk}\left(r=1,2,\cdot \cdot \cdot ,s\right)\\ \lambda_{j}\geq 0,s_{r}^{+}\geq 0,s_{i}^{-}\geq 0\\ j=1,2,\cdot \cdot \cdot ,n\;and\;j\neq k \end{array}\right.$$

where θ is the super-efficiency value, m is the number of input indicators, s is the number of output indicators, and *n* is the number of DMUs. The definitions of the other variables are the same as those described for Equation (1).

#### 3.1.2. Spatial Correlation Test Model

We used Moran’s I index of global spatial autocorrelation [37] to test the spatial correlation of the ECLU:

(3) $$I=\frac{n\sum_{i=1}^{n}\sum_{j=1}^{n}W_{ij}\left(x_{i}-\bar{x}\right)\left(x_{j}-\bar{x}\right)}{(\sum_{i=1}^{n}\sum_{j=1}^{n}W_{ij})\sum_{i=1}^{n}{\left(x_{i}-\bar{x}\right)}^{2}}$$

where *x_i_* is the observed value of region *i*; *n* is the number of regions; *W_i__j_* is the geographic distance weight matrix. The value of Moran’s I is in the range of −1 ≤ I ≤ 1. The closer it is to 1, the higher is the degree of the positive spatial correlation; the closer it is to −1, the higher is the degree of the negative spatial correlation. A value close to 0 indicates that there is no spatial autocorrelation.

#### 3.1.3. Spatial Durbin Model (SDM)

Based on the spatial lag model (SLM) and spatial error model (SEM) [38] considering spatial autocorrelation, Lesage et al. [39] constructed a SDM containing both dependent and explanatory variables. To investigate the externalities of the labor transfer on the ECLU and the ECLU itself, we used the SDM to study the spatial effects of the labor transfer on the ECLU. The SDM is the main model for the determination of the spatial association of geographic elements:

(4) $$\begin{array}{c} TE_{it}=\beta_{0}+\rho \overset{n}{\underset{j=1}{\sum }}W_{ij}TE_{it}+\beta Labor_{it}+\overset{n}{\underset{j=1}{\sum }}\theta W_{ij}Labor_{it}+\beta^{\prime }Labor_{it}^{2}+\overset{n}{\underset{j=1}{\sum }}\theta^{\prime }W_{ij}Labor_{it}^{2}\\ +\alpha X_{it}+\overset{n}{\underset{j=1}{\sum }}\gamma W_{ij}X_{it}+\mu_{i}+\lambda_{t}+\varepsilon_{it} \end{array}$$

where *TE_it_* denotes the ECLU; *Labor_it_* and *Labor_it_*^2^ represent the labor transfer and its squared term (the squared term was added to explore the nonlinear relationship between the labor transfer and cultivated land use efficiency), respectively; *X_it_* is the control variable; $\beta_{0}$ is the constant term; ρ is the spatial autocorrelation coefficient of ECLU; β and $\beta^{\prime }$ are the coefficients of the labor transfer and its squared term, respectively; *W_ij_* is the geographic distance weight matrix; θ, $\theta^{\prime }$, and λ are the coefficients to be estimated; *i* is the region; *t* is the time; $\mu_{i}$ and $\lambda_{t}$ are the spatial and temporal effects, respectively; and $\varepsilon_{it}$ is the random error term.

#### 3.1.4. Spillover Effect

The SDM cannot be used to directly determine the spatial spillover effect. Therefore, we used the partial differential to decompose the SDM. The direct effect obtained from the decomposition represents the effect of the labor transfer in the local region on the ECLU. The indirect effect—that is, the spillover effect—refers to the effect of the local labor transfer on ECLU in adjacent regions. The sum of the direct and indirect effects is the total effect. The corresponding equation has been reported by Le Sage and Pace [39].

### 3.2. Data Sources and Variable Selection

In this study, 31 Chinese provinces (municipalities and autonomous regions, excluding Hong Kong, Macau, and Taiwan) were selected as the research objects. The data were acquired from the China Rural Statistical Yearbook, Finance Yearbook of China, China Statistical Yearbook, Monitoring and Investigation Report on Migrant Workers, and statistical yearbooks of the provinces (municipalities and autonomous regions) for the period of 1991–2019. Based on the zoning by the National Bureau of Statistics in 2011, we divided China’s 31 provinces (municipalities and autonomous regions) into four regions: East, Central, West, and Northeast. The main variables are shown in Table 1.

(1) Dependent variable: ECLU (TE). Based on the research of Chen et al. [40], we selected indicators to calculate the ECLU from the input–output perspective (Table 1). The pollutant generation coefficient, utilization rate, and loss rate of each indicator of cultivated land NPS pollution and the coefficient of each carbon emission indicator were previously reported [41,42,43,44,45];
(2) Independent variable: rural labor transfer (Labor), which is the rural labor transferred from rural areas to urban and secondary and tertiary industries and can be characterized by the ratio of (rural laborers–laborers of rural primary industry) to rural laborers [46];
(3) Control variables: Based on the studies of Li [47] and Dhehibi et al. [48] and considering the data availability, we selected seven indicators as control variables: cultivated land area per laborer (Land), cultivated land multiple cropping index (MCI), peasant’s net income per capita (PINC), irrigation index (IRR), average total power of agricultural machinery (ATP), average agricultural consumption of chemical fertilizers (by effective component, FER), and proportion of agricultural expenditure of the government (AGE).

## 4. Results and Analysis

### 4.1. Rural Labor Transfer and Spatial and Temporal Evolution Characteristics of ECLU

**Figure 1:**
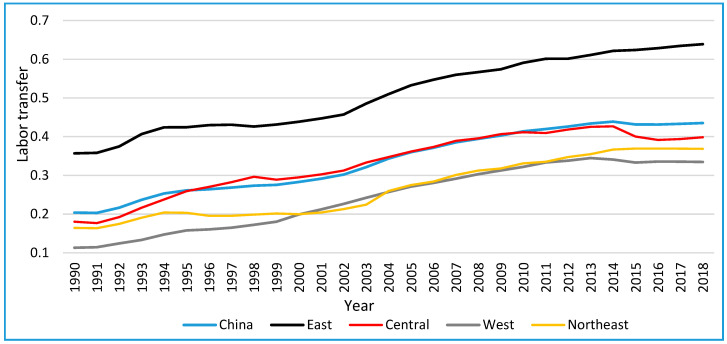
The evolution of the labor transfer from 1990–2018 at the regional and country levels.

Figure 1 shows that China’s rural labor transfer gradually increased from 1990–2013. It reached the maximum of 42.67% in 2014 and started to decrease in 2015. In the study period, the labor transfer showed significant regional differences. The rural labor transfer rate in the east was the highest and reached a peak of 63.90% in 2018, followed by the central, northeastern, and western regions in which the peak values were reached in 2014.

**Figure 2:**
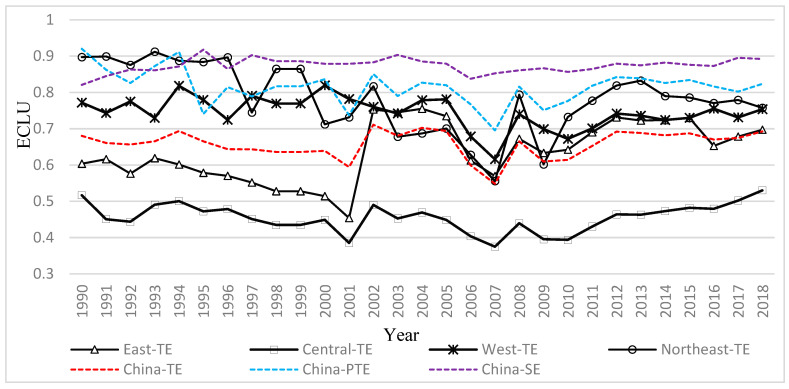
The evolution of the ECLU at the country and regional levels from 1990–2018.

Figure 2 shows the evolution of the TE of the regional and country-level cultivated land use (ECLU), pure technical efficiency (PTE), and scale efficiency (SE) from 1990–2018. The SE of China’s cultivated land use increased, whereas the pure TE exhibited a W-shaped curve over the past 28 years. It gradually flattened after 2009 and started to increase in 2016. With respect to the regional ECLU, China’s ECLU exhibited a repeated trend of increase and decrease, with values ranging between 0.6 and 0.7 from 1990–2018. It decreased to the lowest value (0.53) in 2007 and increased to the highest value (0.71) in 2012. The TE of cultivated land in the east generally decreased from 1990–2001, but it rapidly increased and exceeded the national average after 2001. The TE of cultivated land in Central China was always below the national average. It slightly fluctuated from 1990–2008 and started to slightly increase in 2009. The TE of cultivated land in both the west and northeast always exceeded the national average, but it started to slightly decrease in both regions in 2000.

**Figure 3:**
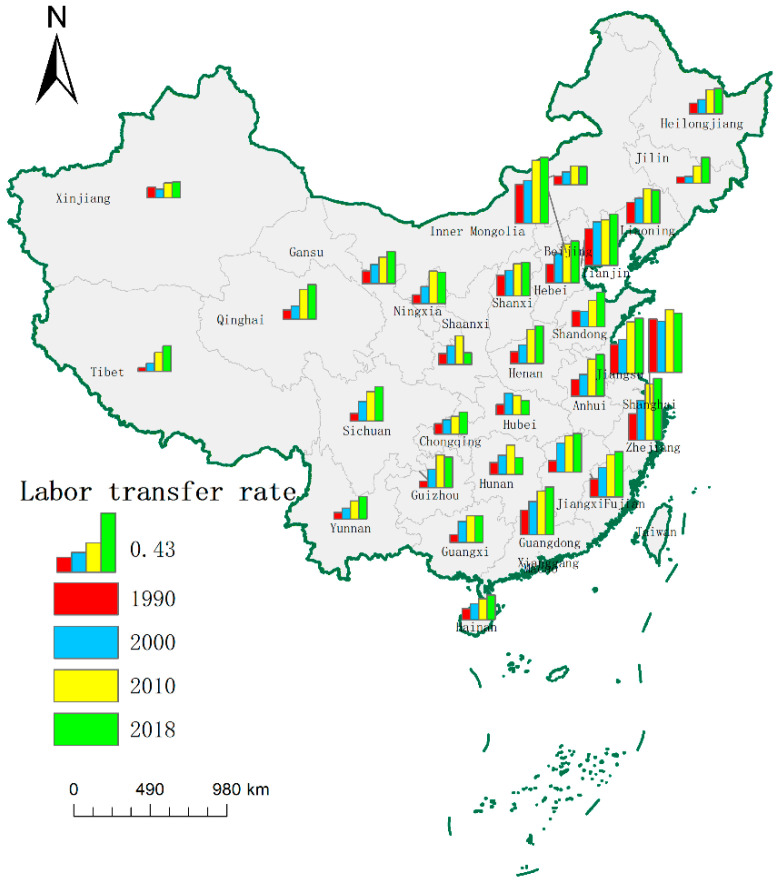
The spatial evolution of the labor transer.

Figure 3 shows the ten provinces (municipalities) in the east, which are situated along the coast. Beijing, Jiangsu, Shanghai, and Zhejiang have the highest labor transfer rate, followed by Tianjin, Hebei, Fujian, and Guangdong. Hainan has the lowest labor transfer rate. In Central China, the Shanxi, Henan, Anhui, and Jiangxi provinces close to the east have higher labor transfer rates than Hunan and Hubei. In the west, the provinces (autonomous regions) with high labor transfer rates are Gansu, Ningxia, Qinghai, Sichuan, and Guizhou. In the northeast, the provinces can be ranked based on the labor transfer rate as follows: Liaoning > Heilongjiang > Jilin. In summary, the labor transfer rate is relatively high in economically developed regions in the east, several central provinces close to the developed regions, and western regions in the upper reaches of the Yellow and Yangtze rivers.

**Figure 4:**
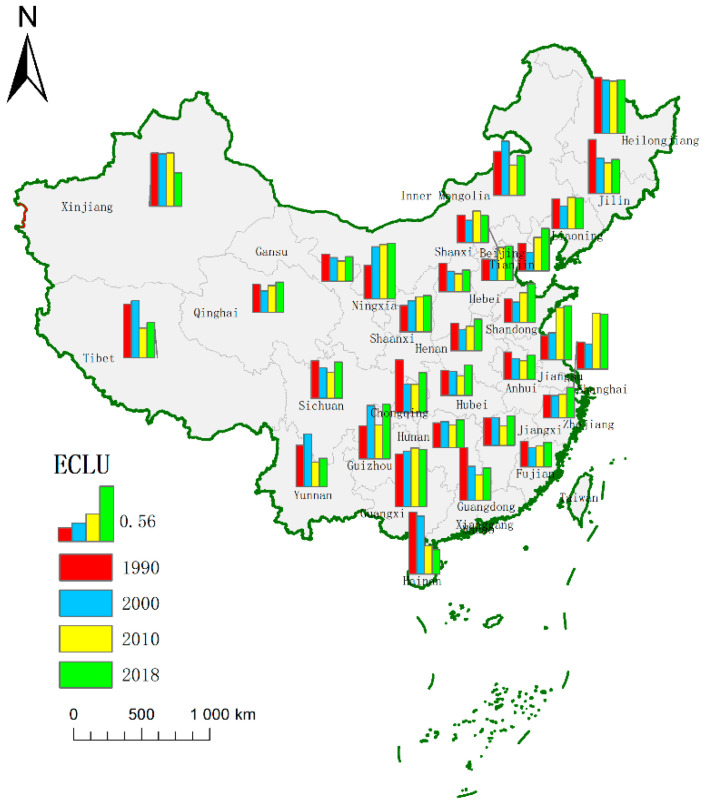
The spatial evolution of the ECLU.

Figure 4 shows that, except for the Hainan Province, the ECLU of Beijing, Shanghai, Tianjin, Hebei, Jiangsu, Zhejiang, and Shandong in the east has been increasing, especially since 2010. In Central China, the ECLU of Hunan, Hubei, and Jiangxi insignificantly changed between 1990 and 2000, but it increased from 1991–2000. The ECLU in most western regions slightly increased after 2000. The ECLU of Xinjiang, Tibet, Inner Mongolia, and the northeastern provinces Jilin and Liaoning significantly fluctuated, but the values of Ningxia and Guangxi were higher.

### 4.2. Spatial Spillover Effect

#### 4.2.1. Spatial Correlation Test

The global Moran’s I index of the ECLU and labor transfer is 0.055 and passes the significance test, indicating a significant spatial correlation between the labor transfer and ECLU (Table 2). From 1990–2006, Moran’s I index showed a significant positive trend; it first increased and then decreased. From 2007–2018, it showed a negative trend; it first increased and then decreased. Moran’s I index changed from positive to negative, although the negative value became smaller and smaller. The spatial correlation between the labor transfer and ECLU remained strong.

#### 4.2.2. SDM Regression Results

The variance inflation factor (VIF) test indicates that the collinearity of the model is not strong. The heteroskedasticity and autocorrelation test imply the lack of heteroscedasticity and autocorrelation of the model. Based on the Hausman test, we selected the fixed effect model. In terms of endogeneity, results of previous studies showed that the SDM itself has certain advantages in dealing with the endogeneity problem due to the omission of variables [49]. Maximum Likelihood (ML) is considered to be an effective method to deal with the endogeneity problem [50]. Elhorst also stated that the spatiotemporal double fixed effect model performs better than the individual fixed effect model [51]. Therefore, we used the spatiotemporal double-fixed Durbin model in combination with ML estimation to analyze the spatial spillover effect of the labor transfer on the ECLU [52]. The model results are shown in Table 3.

At the country level, the coefficients of the labor transfer and its squared term are −0.751 and 1.332, respectively. This means that the labor transfer first negatively affects the ECLU and subsequently the correlation becomes significant and positive. This shows that the correlation between labor transfer and ECLU is not simple and linear but exhibits a “U-shaped” trend. In the early stage of the labor transfer, the effect on environmental pollution increased with the increase in the consumption of chemical fertilizers and pesticides and the labor transfer negatively affects the ECLU. However, the scale and technology effects of cultivated land use have gradually increased since 2009, and the negative effect of labor transfer on the ECLU gradually decreased and eventually changed to a positive effect. From the perspective of other control variables, the cultivated land area per laborer, multiple cropping index, and average total power of agricultural machinery are positively correlated with the ECLU. The irrigation index is negatively correlated with the ECLU in most regions. The consumption of chemical fertilizer correlates with the proportion of the agricultural expenditure of the government, and the effect intensity varies from region to region.

At the regional level, the effect of the labor transfer in the eastern and central regions on the ECLU is insignificant. The east, as economically developed region of China, ranks first with respect to industrial technology and scale, and the scale of labor insignificantly affects the agricultural production. In contrast, the central region is characterized by an underdeveloped economy, land fragmentation, and backward agricultural machinery technology; farmers prioritized the abandonment of land or extensive operation [53,54]. Therefore, the short-term effect of the labor transfer on the ECLU is insignificant. In the west and northeast, the labor transfer and ECLU exhibit a significant U-shaped correlation, indicating that labor transfer promotes the use of technology and scale operation in local agricultural production, reduces the application of chemical fertilizers, increases the application of organic fertilizers and biotechnology, reduces carbon emissions, and thus overall increases the ECLU.

The spatial autoregressive coefficient ρ values of the whole country and the regions are negative. The coefficients of Central, West, and Northeast China are significant at the 1% level, indicating that the labor transfer is negatively correlated with the ECLU of adjacent regions. This shows that the ECLU has a significant negative spatial effect on Central, West, and Northeast China. With respect to the spatial coefficient W, the square term of the labor transfer of the whole country and Central China is positive and significant and the labor transfer coefficients of West and Northeast China are negative and significant, indicating that the spatial spillover effect of labor transfer on the ECLU in the whole country, Central, West, and Northeast is significant. To clarify the differences with respect to the effects of the labor transfer in local and other regions on the ECLU, the ECLU was further decomposed. The results are shown in Table 4.

#### 4.2.3. Effect Decomposition

As indicated in Table 4, at the country level, the direct effect of the labor transfer on the ECLU is significantly negative. The correlation curve displays a U-shaped trend; that is, the correlation is first negative and then positive (the coefficients of *Labor* and *Labor*^2^ are −0.740 and 1.314, respectively). With respect to the indirect effect, the coefficient of *Labor*^2^ is 0.962 and significant at the 1% level, indicating that there exists a spatial spillover effect of the labor transfer on the ECLU. These findings are consistent with H1.

At the regional level, the direct effect of the labor transfer on the ECLU is insignificant in the east region. The labor transfer coefficients of Central and West China are 0.540 and −1.456, respectively, and both pass the significance test. The square terms of the labor transfer in the west and northeast regions are both significant at the 5% level, indicating that the positive effects of Central and Northeast China are more significant, and the changes due to the labor transfer in the West are more significant. In terms of the indirect effect, both Central and Northeast China have significant negative spatial spillover effects. However, after a certain labor transfer in the central region, the spatial spillover effect becomes positive. In Central and Northeast China, the influence of the labor transfer on the ECLU had spatial spillover effects. In the east and west regions, the effect was insignificant, possibly because the eastern coastal cities belong to provinces with a large labor input, do not have large agricultural production areas, and changes in the local agricultural input insignificantly affect the main grain-producing areas in surrounding large agricultural production areas. Also, various agricultural machinery technologies and farming modes are mostly disseminated in the central and western regions based on the return of rural labor. The western region is far away from the economically developed regions of the east. Labor transfer mainly occurs within the respective province, and laborers can easily obtain concurrent work. These findings are consistent with H2.

In terms of control variables, the cultivated land area per laborer and multiple cropping index positively and significantly affect the ECLU in the east and areas adjacent to Central and West China. The increase in the cultivated land area per laborer benefits large-scale operations, and crops with different multiple cropping indices can absorb harmful elements that are not fully utilized by the soil. The peasant’s net income per capita positively and significantly affects the ECLU in East, Central, and Northeast China, but an opposite trend can be observed in the west. With respect to the indirect effect, the peasant’s net income per capita in the west and northeast is positive and significant, indicating that it has a significant positive external effect on the ECLU. The irrigation index has a negative effect on ECLU, which is consistent with the findings of many scholars but seems counterintuitive. The irrigation index reflects the proportion of the water coverage rather than the irrigation efficiency. The negative effect of the irrigation index on the ECLU indicates that farmland water conservancy facilities in most regions of China should be strengthened to facilitate scientific irrigation and drainage. The total power of agricultural machinery in adjacent areas can effectively improve the ECLU in Eastern and Northeast China. The average fertilizer application in the east is not conducive to its ECLU, but the situation is opposite in the central and northeast. The ECLU in Western and Northeast China is significantly affected by the amount of average chemical fertilizer applied in their neighboring areas. The action directions of the proportion of the agricultural expenditure of the government are consistent in local and adjacent regions and mainly correlate with the consumption of chemical fertilizers and pesticides.

### 4.3. Robustness Test

To test the robustness of the empirical conclusions, we used the adjacency and economic distance matrices instead of the geographic distance matrix to conduct an empirical test at the country level (Table 5). Based on the test results, both models are significant at the 1% level, and the coefficients of the key variables are consistent with the coefficients and action direction of the geographic distance matrix, indicating that conclusions regarding the effect of rural labor transfer on the ECLU and the spatial spillover effect are very robust.

## 5. Discussion

Based on the continuous transfer of rural labor in China and the transformation of the efficiency and methods of cultivated land use (especially the significant increase in the pressure on the food and vegetable supply due to the global COVID-19 epidemic), farmers’ conditions and land use efficiency are important issues of sustainable development in China and worldwide. In this study, the mechanism based on which rural labor transfer affects the ECLU and the spatial spillover effect were analyzed. The focus was mainly placed on the spatiotemporal changes and spatial spillover characteristics of the ECLU in the whole country and different regions due to rural labor transfer in China. We used the super-efficiency EBM model to calculate the ECLU, which is more accurate than the super-efficiency SBM model. We also used the spatiotemporal double-fixed SDM model to explore the correlation between the labor transfer and ECLU, as well as the spatial spillover effect of rural labor transfer on the ECLU.

At the country level, the spatial spillover effect of China’s rural labor transfer on the ECLU first increases and then decreases. It does not negatively affect the ECLU. At the regional level, the spatial spillover effect caused by the rural labor transfer in the central and northeastern regions has an antagonistic effect on the ECLU. The ECLU in rural areas of the central region first increases and then decreases. Zeng et al. [55] studied the overall characteristics of the eco-efficiency of agricultural production in China and reported that it declined in the early 21st century and then continued to increase, which is basically consistent with the evolution of the ECLU in each region observed in this study.

The most Important conclusion of this study is that the indirect effect is the basis for the determination that the labor transfer in Central, West, and Northeast China is negatively correlated with the ECLU in adjacent regions. However, in several previous studies, the criterion for the determination of the spatial spillover effect was generally considered to be the ρ value of the regression results of the SDM model [39]. For example, when the spatial spillover effects of the transportation infrastructure on urban industries were analyzed, Xie et al. [56] separately discussed the direct and indirect spatial effects. However, we believe that the output of the spatial econometric model has spatial characteristics, and thus it is necessary to analyze the spatial effects from three aspects: direct, indirect, and total effects. Chen and Chi [57] also mentioned that the focus should not exclusively be placed on the influence coefficients of the regression results of the spatial econometric model, but attention should be paid to the direct, indirect, and total effects of independent variables on dependent variables.

We used the statistical data of the period 1990–2018 to calculate the ECLU. In this period, the trend of the labor outflow was relatively stable. Therefore, investigating the use behavior of farmers’ cultivated land has a relatively stable reference value. However, the rural labor flow and cultivated land use behavior have been subject to changes since 2019 due to the COVID-19 pandemic. The rural labor flow, efficiency of cultivated land use, methods of cultivated land use, and spatial spillover effect from 2019–2022 must be studied in depth.

## 6. Conclusions

We used the undesirable super-efficiency EBM model and SDM to analyze the effect of rural labor transfer on the ECLU and its spatial spillover effect in China at the regional and country levels for the period 1990–2018. The results imply the following: (1) China’s rural labor transfer rate generally increased. The rural labor transfer rate was higher in the east, provinces close to the east, and in the west in the upper reaches of the Yellow and Yangtze rivers. A return of rural labor has been observed in most regions since 2015. (2) The ECLU in China has repeatedly increased and decreased. In eastern provinces (municipalities), the ECLU has increased and exceeded the national average since 2001. In most areas in Central China, the ECLU increased from 1991–2000 but remained below the national average. In most areas in the west and northeast, the ECLU was higher. (3) The SDM results indicate that the effect of China’s labor transfer on the ECLU was first negative and then positive (U-shaped curve). The change from the use of chemical fertilizers to biotechnology and modern agricultural machinery technology is key to the change of the ECLU. The effect of rural labor transfer on the ECLU in the west and northeast follows a significant U-shaped trend, but this effect was insignificant in East and Central China. (4) With respect to the effect decomposition, at the country level, the impact of labor transfer on the ECLU shows a significant U-shaped change, and the impact of labor transfer of neighboring areas on the ECLU is still U-shaped change, but it is not completely significant. Both Northeast and Central China had significantly negative spatial spillover effects, and the spatial spillover effect of Central China followed a U-shaped trend.

## 7. Policy Recommendations

Based on the above conclusion, the science and technology, cultivated land protection, and farmland use policy of regions or countries with different degrees of economic development have big differences, so the study obtains the following enlightenment: (1) Based on advanced modern agricultural production technology, economic developed areas and countries with fewer people and more land should give full play to a positive space spillover effect by enhancing cultivated land use intensive, promoting the scale benefit, so as to form a benign interaction to other surrounding countries and regions. (2) For Central China or other countries with relatively low production technology efficiency, first of all, the training of agricultural labor skills should be strengthened, and their ability and willingness to use modern agricultural production technology should be enhanced. Secondly, these countries should actively promote the farmland leveling project, and the transfer of agricultural land; improve the degree of intensive utilization of cultivated land; enhance the feasibility of using modern farming technology for production, so as to promote the synchronous growth of the technical efficiency and scale efficiency of cultivated land utilization. (3) In view of significant space negative externalities and regional differences, it is recommended to encourage farmers to use biotechnology, organic fertilizers produced by animals, and plants replacing chemical fertilizers to reduce the pollution emissions, strengthen the common governance between regions, and avoid negative effects of pollution transfer due to improper local pollution control aiming at increasing the global ECLU through scientific fertilizer application and large-scale production. (4) For developing countries with more people and less land, especially the countries with greater rural populations, they should more actively promote the centralized construction of residential areas and improve the layout of rural villages through reclamation, idle homestead replacement, withdrawing villages and building towns, and then reducing the occupation of cultivated land. In the planning process, we should always take the economical and intensive land use as the planning goal, and then greatly increase the area of cultivated land. At the same time, it is essential to strengthen the development and aggregation of construction land within the village, and optimize the layout of various construction land.

## Figures and Tables

**Table 1:** A descriptive statistical analysis of variables.

Indicator	Variable Name	Indicator	Variable Name
Input indicators	Agricultural laborers (10^4^ people)	Dependent variable	ECLU (*TE*)
	Total sown area of farm crops (10^3^ hectares)	Independent variables	Labor transfer (*Labor*; %)
	Total power of agricultural machinery (10^4^ kW)		Squared term of labor transfer (*Labor*^2^; %)
	Effective irrigation area/10^3^ hectares	Control variables	Cultivated land area per laborer (*Land*; hectare)
	Agricultural consumption of chemical fertilizers by effective component (10^4^ tons)		Multiple cropping index (*MCI*; %)
	Consumption of pesticides (10^4^ tons)		Peasant’s net income per capita (*PINC*; yuan)
	Consumption of agricultural plastic film (10^4^ tons)		Irrigation index (*IRR*; %)
Desirable output indicators	Gross crop production (10^4^ tons)		Average total power of agricultural machinery (*ATP*; kW)
Undesirable output indicators	Carbon emissions (10^4^ tons)		Average agricultural consumption of chemical fertilizers (by effective component, *FER*; ton)
	NPS pollution (10^4^ tons)		Proportion of agricultural expenditure of government (*AGE*; %)

**Table 2:** Bivariate global Moran’s I.

Year	Moran’s I	Year	Moran’s I
1990	0.188 ***	2005	0.071 **
1991	0.191 ***	2006	0.014
1992	0.198 ***	2007	−0.068
1993	0.051	2008	−0.158
1994	0.011	2009	−0.155
1995	0.019 *	2010	−0.181
1996	0.107 ***	2011	−0.182
1997	0.127 ***	2012	−0.192
1998	0.101 ***	2013	−0.157
1999	0.148 ***	2014	−0.149
2000	0.199 ***	2015	−0.124
2001	0.201 ***	2016	−0.075
2002	0.126 ***	2017	−0.092
2003	0.071 **	2018	−0.089
2004	0.127 ***	1990–2018	0.055 ***

Note: *, **, and *** indicate significance at the 10%, 5%, and 1% levels, respectively.

**Table 3:** The SDM regression results for the whole country and individual Chinese regions.

Variables	Whole Country	East	Central	West	Northeast
*Labor*	−0.751 ***	−0.668	0.16	−1.825 ***	−3.955 *
*Labor* ^2^	1.332 ***	0.992	0.248	3.181 ***	4.530 *
*Land*	0.174 **	1.222 ***	0.311 *	0.260 *	−0.00639
*MCI*	0.236 ***	0.448 ***	0.179 ***	0.159 ***	−0.562
*Ln.PINC*	−0.00791	0.277 *	0.233 ***	−0.035	1.717 ***
*IRR*	−0.345 ***	−0.201	−0.515 ***	−0.399 **	−2.512 ***
*ATP*	0.00864 **	0.0219 ***	−0.00237	0.000971	0.201 **
*FER*	−0.293 ***	−0.862 ***	0.142	−0.361 *	7.284 ***
*AGE*	−0.846 **	0.441	−2.766 ***	−2.326 ***	4.198 **
*W* × *Labor*	−0.488	−0.807	−1.681	−6.591 *	−7.833 **
*W* × *Labor*^2^	1.089 ***	0.192	3.656 *	9.095	4.946
*ρ*	−0.0583	−0.0585	−1.196 ***	−0.974 ***	−0.833 ***
*Sigma*	0.0157 ***	0.0108 ***	0.0004 ***	0.0110 ***	0.0010 ***

Note: *, **, and *** indicate significance at the 10%, 5%, and 1% levels, respectively.

**Table 4:** Effect decomposition.

	Variables	Whole Country	East	Central	West	Northeast
Direct effect	*Labor*	−0.740 ***	−0.639	0.540 *	−1.456 **	−1.828
	*Labor* ^2^	1.314 ***	0.956	−0.433	2.716 **	3.920 **
	*Land*	0.178 **	1.279 ***	0.422 ***	−0.065	0.0241
	*MCI*	0.236 ***	0.450 ***	0.141 ***	0.0557	−0.241
	*Ln.PINC*	−0.0146	0.268 *	0.247 ***	−0.189 *	1.266 ***
	*IRR*	−0.341 ***	−0.179	−0.398 ***	−0.337 **	−1.254 **
	*ATP*	0.00844 **	0.0208 ***	−0.00122	−0.00393	0.0457
	*FER*	−0.291 ***	−0.863 ***	0.141 **	−0.0815	4.669 ***
	*AGE*	−0.810 **	0.525	−1.812 ***	−1.868 ***	3.560 ***
Indirect effect	*Labor*	−0.42	−0.644	−1.198 *	−2.702	−4.494 **
	*Labor* ^2^	0.962 ***	−0.0711	2.144 **	3.259	1.028
	*Land*	0.345	−1.831	−0.32	2.605 ***	−0.0409
	*MCI*	0.071	−0.0907	0.129	0.801 ***	−0.665
	*Ln.PINC*	0.668 ***	0.479	−0.0384	1.172 **	0.967 *
	*IRR*	−0.0053	−1.919 ***	−0.376 *	−0.365	−2.760 ***
	*ATP*	0.0158 *	0.123 ***	−0.00418	0.0356	0.339 **
	*FER*	−0.410 ***	−0.122	−0.000534	−2.217 ***	5.544 ***
	*AGE*	−1.320 *	4.594	−2.917 ***	−3.18	1.675
Total effect	*Labor*	−1.160 ***	−1.283	−0.658	−4.158 **	−6.322 *
	*Labor* ^2^	2.276 ***	0.885	1.711	5.975 *	4.948
	*Land*	0.523 **	−0.552	0.102	2.540 ***	−0.0168
	*MCI*	0.307 ***	0.359 *	0.270 **	0.857 ***	−0.906
	*Ln.PINC*	0.654 ***	0.747	0.208	0.983 **	2.233 ***
	*IRR*	−0.346 *	−2.099 ***	−0.774 ***	−0.702	−4.013 ***
	*ATP*	0.0242 ***	0.144 ***	−0.00541	0.0317	0.385 **
	*FER*	−0.702 ***	−0.985 **	0.14	−2.299 ***	10.21 ***
	*AGE*	−2.130 ***	5.119	−4.729 ***	−5.048 ***	5.235 *

Note: *, **, and *** indicate significance at the 10%, 5%, and 1% levels, respectively.

**Table 5:** A robustness test at the country level.

Variables	Adjacency Matrix	Direct Effect	Indirect Effect	Total Effect	Economic Matrix	Direct Effect	Indirect Effect	Total Effect
*Labor*	−0.751 ***	−0.740 ***	−0.42	−1.160 ***	−0.576 ***	−0.581 ***	−0.576 **	−1.157 ***
*Labor* ^2^	1.332 ***	1.314 ***	0.962 ***	2.276 ***	1.196 ***	1.217 ***	1.443 ***	2.659 ***
*Land*	0.174 **	0.178 **	0.345	0.523 **	0.135 *	0.146 **	0.230 **	0.376 ***
*MCI*	0.236 ***	0.236 ***	0.071	0.307 ***	0.235 ***	0.233 ***	−0.105 ***	0.128 ***
*Ln.PINC*	−0.00791	−0.0146	0.668 ***	0.654 ***	−0.0563	−0.0463	0.506 ***	0.460 ***
*IRR*	−0.345 ***	−0.341 ***	−0.0053	−0.346	−0.307 ***	−0.299 ***	0.159	−0.14
*ATP*	0.00864 **	0.00844 **	0.0158 *	0.0242 ***	0.0121 ***	0.0121 ***	0.00335	0.0154 ***
*FER*	−0.293 ***	−0.291 ***	−0.410 ***	−0.702 ***	−0.460 ***	−0.462 ***	0.0353	−0.427 ***
*AGE*	−0.846 **	−0.810 **	−1.320 *	−2.130 ***	−1.153 ***	−1.085 ***	1.896 ***	0.811
*W* × *Labor*	−0.488				−0.513 **			
*W* × *Labor*^2^	1.089 ***				1.313 ***			
*sigma*	0.0157 ***				0.0141 ***			

Note: *, **, and *** indicate significance at the 10%, 5%, and 1% levels, respectively.

## Data Availability

The data presented in this study are available on request from the corresponding author.
